# Exogenous Gibberellin Delays Postharvest Leaf Senescence in Pak Choi by Modulating Transcriptomic and Metabolomic Profiles

**DOI:** 10.3390/foods14060981

**Published:** 2025-03-13

**Authors:** Dan Wang, Xiuyun Zhao, Tongbing Su, Weihong Wang, Xiaoyun Xin, Bin Zhang, Deshuang Zhang, Yangjun Yu, Zhongjiang Wang, Fenglan Zhang, Linyi Zhou, Peirong Li, Shuancang Yu

**Affiliations:** 1 School of Food and Health, Beijing Technology and Business University, Beijing 100048, China; nwangdan@126.com; 2 Beijing Vegetable Research Center (BVRC), Beijing Academy of Agriculture and Forestry Sciences (BAAFS), Beijing 100097, China; zhaoxiuyun@nercv.org (X.Z.); sutongbing@nercv.org (T.S.); wangweihong@nercv.org (W.W.); xinxiaoyun@nercv.org (X.X.); brzhangbin@nercv.org (B.Z.); zhangdeshuang@nercv.org (D.Z.); yuyangjun@nercv.org (Y.Y.); zhangfenglan@nercv.org (F.Z.); yushuancang@nercv.org (S.Y.); 3 State Key Laboratory of Vegetable Biobreeding, Beijing Vegetable Research Center (BVRC), Beijing Academy of Agriculture and Forestry Sciences (BAAFS), Beijing 100097, China; 4 Key Laboratory of Biology and Genetic Improvement of Horticultural Crops (North China), Ministry of Agriculture, Beijing 100097, China; 5 Beijing Key Laboratory of Vegetable Germplasm Improvement, Beijing 100097, China; 6 College of Food Science, Northeast Agricultural University, Harbin 150030, China; wzjname@126.com

**Keywords:** gibberellin, chlorophyll degradation, leaf senescence, storage, pak choi

## Abstract

Postharvest leaf senescence is a pivotal determinant influencing the quality and shelf life of leafy vegetables, exemplified by pak choi (*Brassica rapa* L. subsp. *chinensis*). While the regulatory role of gibberellin (GA) in modulating leaf senescence has been documented across diverse plant species, the underlying physiological and molecular mechanisms remain insufficiently characterized. This study, through a combination of transcriptomic and metabolomic analyses, investigated the effect of exogenous GA on postharvest leaf senescence in pak choi. GA treatment alleviated etiolation, maintained chlorophyll levels, reduced conductivity and malondialdehyde content, and delayed the onset of senescence symptoms in postharvest pak choi. Transcriptome profiling indicated that GA suppressed the expression of the senescence-associated genes *BraSRGs* and *BraSAGs*. In addition, GA influenced chlorophyll degradation and preserved chlorophyll content by modulating the expression of genes implicated in chlorophyll metabolism, including *BraPPH*, *BraSGR1*, *BraNYCI*, and *BraPAO*. GA treatment impacted lipid levels and regulated the degradation of membrane phospholipids. Furthermore, exogenous GA treatment disrupted the efficacy of the jasmonic acid signal pathway, primarily through the transcriptional downregulation of key regulatory genes, including *BraJAZ10* and *BraJAR1*. These results provide insights into the role of GA in delaying postharvest leaf senescence and highlight potential targets for improving postharvest management in leafy vegetables.

## 1. Introduction

Pak choi (*Brassica rapa* L. subsp. *chinensis*) is a widely consumed leafy vegetable valued for its rich nutritional profile, including vitamins, minerals, antioxidants, and reported anti-cancer properties [1]. Despite its nutritional benefits, pak choi is highly perishable due to rapid postharvest senescence, which manifests as leaf yellowing, wilting, and decay, resulting in substantial economic losses during storage, transportation, and retail distribution [2]. Therefore, developing effective postharvest preservation strategies is crucial for maintaining pak choi’s quality and extending its shelf life.

Leaf senescence, the terminal phase of leaf development, is a tightly regulated process influenced by both developmental cues and environmental factors, including postharvest stresses like detachment [3]. The rapid onset of senescence in harvested leafy vegetables, often exacerbated by nutrient depletion and hormonal changes [4], leads to significant quality losses. Several plant growth regulators have demonstrated efficacy in delaying senescence. For instance, 6-benzyladenine has been shown to enhance antioxidant capacity and preserve chlorophyll [5], while salicylic acid modulates phenylpropanoid metabolism and ROS homeostasis [6]. Melatonin has been reported to influence flavonoid biosynthesis and the expression of related genes (e.g., *BrFLS1* and *BrFLS3.2*) and to suppress genes associated with chlorophyll degradation and abscisic acid production [7,8]. Despite these advances, a comprehensive understanding of the molecular mechanisms by which these regulators influence postharvest senescence in leafy vegetables is still lacking, highlighting the need for further investigation.

Gibberellin (GA), a class of phytohormones belonging to the five major plant hormone groups, are central regulators of multiple developmental processes, including seed germination, stem elongation, and floral transition [9]. In recent years, research has focused on the role of GA in delaying the senescence of postharvest fruits and vegetables. Studies on kiwifruit [9], okra [10], and tomato [11] have demonstrated that GA application can effectively delay the ripening process and enhance postharvest preservation. In particular, GA has been shown to antagonize the effects of abscisic acid by restraining oxidative stress, reducing lipid peroxidation, and slowing the degradation of pigments and soluble proteins [12]. Furthermore, GA treatment has been found to contribute to chlorophyll retention and delay senescence in citrus fruits during storage, primarily by modulating the metabolism of chlorophyll and carotenoids [13]. Recent studies have also found that GA affected the expression of several key genes associated with senescence, including *BrNAC087*, *BrPPH*, and *BrRCCR*, in postharvest cabbage [14]. In comparison with other phytohormones, gibberellin exerts a more direct influence on the growth, development, and preservation of fruits. Gibberellin not only impacts cell division but also modulates diverse physiological and metabolic processes in plants. For instance, it promotes the hydrolysis of starch and regulates respiration, thereby affecting the rate of energy consumption and the overall physiological state of the fruit [15]. Additionally, the existing research on the application of GA in postharvest pak choi is still relatively scarce and the comprehensive mechanism by which GA affects senescence at the molecular level, especially in leafy vegetables, remains unclear.

In summary, while significant advances have been made in understanding the hormonal regulation of postharvest senescence, the molecular mechanisms, especially those involving GA, remain to be fully elucidated. This study aims to investigate the impact of GA application on postharvest leaf senescence in pak choi, focusing on the molecular pathways that govern this process. By examining changes in gene expression and metabolite profiles, this research seeks to provide deeper insights into the role of GA in maintaining the quality and shelf life of leafy vegetables, with potential applications for postharvest management and commercial production.

## 2. Materials and Methods

### 2.1. Plant Materials and Samples

The plant materials utilized in this study were sourced from the Beijing Academy of Agricultural and Forestry Sciences, China. The plant materials were harvested after 40 days of sowing and transported to the laboratory within one hour to ensure minimal deterioration. Samples were selected based on uniform size (20–35 g), vibrant green color, absence of disease or pests, and no visible mechanical damage. Preliminary Experiment: the samples were treated with GA_3_ (Biotopped Technology Co., Ltd., Beijing, China) solutions at concentrations of 0 mg L^−1^, 25 mg L^−1^, 50 mg L^−1^, and 100 mg L^−1^. Following a 5-day storage period, fundamental quality parameters were assessed. Main Experiment: Based on the preliminary results, the newly acquired samples were systematically allocated into two distinct experimental cohorts. One was subjected to a treatment with 100 mg L^−1^ GA_3_ solution for 20 min, referred to as the gibberellin-treated group (GA). The other was treated with water for the same duration and served as the control group (C). Following treatment, six pak choi samples were individually packaged in 0.03 mm polyethylene film bags, with a total of 30 bags prepared for each group. All samples were stored at 20 ± 0.5 °C and 80–90% relative humidity environment. The storage environment was regulated using an RDN-600 Climate Control Device (Ningbo Yanghui Instrument Co., Ltd., Ningbo, China), internally equipped with high-precision sensors to continuously monitor temperature at regular intervals. Physiological indices were measured from the outermost leaves, avoiding the main vein, at 2 d intervals during storage. For gene expression analyses, leaf samples were snap-frozen in liquid nitrogen and stored at −80 °C for subsequent gene expression and metabolite abundance analyses.

### 2.2. Measurement of Weight Loss, Leaf Color, and Relative Conductivity

Uniform-sized samples from both experimental groups were weighed at each time point to assess weight, and the weight loss rate was calculated accordingly. Leaf color was measured using a CR-400 colorimeter (Konica Minolta, Tokyo, Japan), which recorded the lightness (L), redness-greenness (a), and yellowness-blueness (b) parameters. Each leaf was measured at six equidistant positions and averaged. The hue angle (H) was calculated as follows: H = tan^−1^ (b/a), as mentioned by Lande et al. [16]. An increase in the L value and a decrease in the H value correlate with more pronounced yellowing of the leaves. Leaf relative conductivity was measured using a DDSJ-308A conductivity meter (Inesa, Shanghai, China) following the method described by Wang et al. [17]. Tissue discs with uniform thickness (20 discs, approximately 1 g) were soaked in 20 mL of distilled water for 12 h, and the solution conductivity was measured (R_0_). Then, the entire setup was placed in a boiling water bath for 30 min. After cooling, the electrical conductivity (R_1_) was measured again. The relative conductivity was determined using the following formula: Relative conductivity = R_0_/R_1_ × 100%.

### 2.3. Determination of Chlorophyll Content

Chlorophyll content was determined following the method described by Wang et al. [18], with minor modifications. Briefly, 1.0 g of leaf tissue was extracted with 10 mL of 95% ethanol. The mixture was centrifuged at 10,000× *g* for 3 min at 4 °C, and the supernatant was collected. The absorbance was measured at 649 and 665 nm using a L6S spectrophotometer (Inesa, Shanghai, China).

### 2.4. Measurement of Malondialdehyde (MDA) Content

The content of malondialdehyde (MDA), a marker of oxidative stress, was determined using the thiobarbituric acid (TBA) assay method as described by Ohkawa et al. [19]. Leaf samples (0.1 g) were homogenized in 1 mL of 10% trichloroacetic acid (TCA) and centrifuged at 12,000× *g* for 10 min at 4 °C. A 200 μL aliquot of the supernatant was mixed with 300 μL of 0.67% TBA, and the mixture was reacted for 30 min at 100 °C. After cooling, the absorbance was measured at 532 nm and 600 nm using a microplate reader (Molecular Devices, San Jose, CA, USA).

### 2.5. Determination of Soluble Sugar Content

The soluble sugar content was quantified using a Plant Soluble Sugar Content Assay Kit (Beijing Boxbio Science and Technology Co., Ltd., Beijing, China) based on the anthrone colorimetric method. Briefly, 0.1 g of frozen leaf tissue was homogenized and then heated at 95 °C for 10 min. After cooling, the sample was centrifuged at 12,000× *g* for 10 min. A total of 40 μL of the sample supernatant, 40 μL of distilled water, 20 μL of anthrone reagent, and 200 μL of concentrated sulfuric acid were mixed. A standard glucose solution series (0.3, 0.2, 0.1, 0.05, 0.025, and 0.0125 g L^−1^) was prepared to establish a standard curve. The reaction mixture was then heated at 95 °C for 10 min, and, after cooling, the absorbance was measured at 620 nm. The soluble sugar content was calculated using the standard equation derived from the glucose standard curve.

### 2.6. Chlorophyllase Activity Assay

Chlorophyllase activity was measured using a plant chlorophyllase ELISA kit (Mlbio, Shanghai, China). A standard curve was generated by plotting the enzyme activity (kU L^−1^) against absorbance using linear regression. The sample chlorophyllase activity was calculated by comparing its absorbance with the standard curve.

### 2.7. RNA Sequencing and Data Analysis

Total RNA was extracted from pak choi leaves at different time points: the harvest day (0d), four days postharvest for both control (C-4d) and GA-treated (GA-4d) groups, and ten days postharvest for control (C-10d) and GA (GA-10d) groups. The RNA was extracted using the RNAprep Pure polysaccharide polyphenol plant total RNA extraction kit (TIANGEN, Beijing, China). Sequencing libraries were constructed using the NEBNext^®^ Ultra™ II RNA Library Prep Kit for Illumina^®^ sequencing (NEB, Ipswich, MA, USA). Libraries were sequenced on an Illumina platform by Metware Biotechnology Co., Ltd. (Wuhan, China). Raw sequencing data were processed using Feature Counts to calculate gene alignment, and FPKM values for each gene were calculated based on gene length.

### 2.8. Identification of Differentially Expressed Genes (DEGs)

Differentially expressed genes (DEGs) were identified by comparing gene expression levels between the GA-treated and control groups at corresponding time points. DESeq2 was used to analyze the differential expression of genes, and the *p*-values were corrected using the Benjamini and Hochberg method. The threshold for significant DEGs was set to |Fold Change| > 1.5 and *p* < 0.05. Functional enrichment analysis of the DEGs was performed using the hypergeometric test. Gene Ontology (GO) and Kyoto Encyclopedia of Genes and Genomes (KEGG) pathway analyses were conducted to identify enriched terms and pathways related to the DEGs.

### 2.9. Metabolite Analysis

Leaf samples were freeze-dried and subsequently ground into powder. A total of 50 mg of the sample was extracted with 70% methanol. The mixture was centrifuged at 12,000× *g* for 3 min, and the supernatant was filtered through a 0.22 μm membrane and stored in an injection bottle for subsequent analysis. The sample extracts were analyzed using a UPLC-ESI-MS/MS system (ExionLC™ AD, Sciex, Framingham, MA, USA). Agilent SB-C18 column (1.8 µm, 2.1 mm × 100 mm) was used. The mobile phase consisted of solvent A (0.1% formic acid in pure water) and solvent B (0.1% formic acid in acetonitrile). Elution program: 0–9 min, 95% A to 5% A; 9–10 min, maintained at 5% A; 10–11.1 min, returned to 95% A; and 11.1–14 min, re-equilibration at 95% A. The flow rate was 0.35 mL/min. The column temperature was 40 °C. The injection volume was 2 μL. The ESI source temperature was set to 500 °C with ion spray voltage of 5500 V (positive ion mode) and −4500 V (negative ion mode). Ion source gas I, gas II, and curtain gas were set to 50, 60, and 25 psi, respectively. Collision-activated dissociation (CAD) was set to high, with nitrogen as the collision gas.

Metabolite quantification was performed using the multiple reaction monitoring (MRM) mode based on the Metware database (Metware Biotechnology Co., Ltd.). Raw mass spectrometry data were processed using MultiQuant software (version 3.0.3, Sciex, Framingham, MA, USA), which included baseline filtering, peak identification, integration, and retention time correction. The peak area of each chromatographic peak represented the relative abundance of the corresponding metabolite.

Quality control (QC) samples were prepared by pooling equal volumes of extracts from all samples, which were injected at regular intervals to monitor the stability and reproducibility of the analytical system. Orthogonal partial least squares discriminant analysis (OPLS-DA) was performed using the R package MetaboAnalystR (version 4.0.0) to identify significant metabolites based on Variable Importance in Projection (VIP) scores. Differentially accumulated metabolites (DAMs) were identified using VIP score (VIP > 1) and |Fold Change| > 1.5. Metabolites were annotated based on the KEGG compound database (http://www.kegg.jp/kegg/compound/, accessed on 28 June 2024).

### 2.10. Visualized Analysis of DEGs and DAMs

The visualization analysis of transcriptional and metabolic results includes the following components: The HCA (hierarchical cluster analysis) results were presented as heatmaps with hierarchical clustering, and were conducted using the R package ComplexHeatmap (version 2.20.0). Normalized signal intensities of metabolites (Z-score) are visualized as a color spectrum, which represents the relative abundance of metabolites, with red indicating high abundance and blue indicating low abundance. Volcano plots were generated to visualize the significance and fold changes of DEGs and DAMs. The plots were created using the R package ggplot2 (version 3.4.4). The screening criteria for DEGs were set as |Fold Change| > 1.5 and adjusted *p*-value (FDR) < 0.01. For DAMs, the criteria were |Fold Change| > 1.5 and VIP score > 1. Venn diagrams were used to illustrate the overlap of DEGs or DAMs across different comparison groups. The diagrams were generated using the online tool jvenn (http://jvenn.toulouse.inra.fr/, accessed on 14 November 2024). The enrichment results were visualized as bubble charts using the Canva tool (https://www.canva.com/, accessed on 25 November 2024). The degree of enrichment was measured by the Rich factor, q-value, and the number of genes enriched in each pathway. Pathways with a q-value < 0.05 were considered significantly enriched.

### 2.11. Statistical Analysis

All experiments were performed with three biological replicates, and each measurement was repeated three times. The statistical analyses were conducted using SPSS software (version 17.0). The results are presented as mean ± standard error (SE), and significant differences between groups were assessed using ANOVA followed by Tukey’s post hoc test (*p* < 0.05 or *p* < 0.01).

## 3. Results

### 3.1. Effect of GA Treatment on the Quality of Postharvest Pak Choi

**Figure 1:**
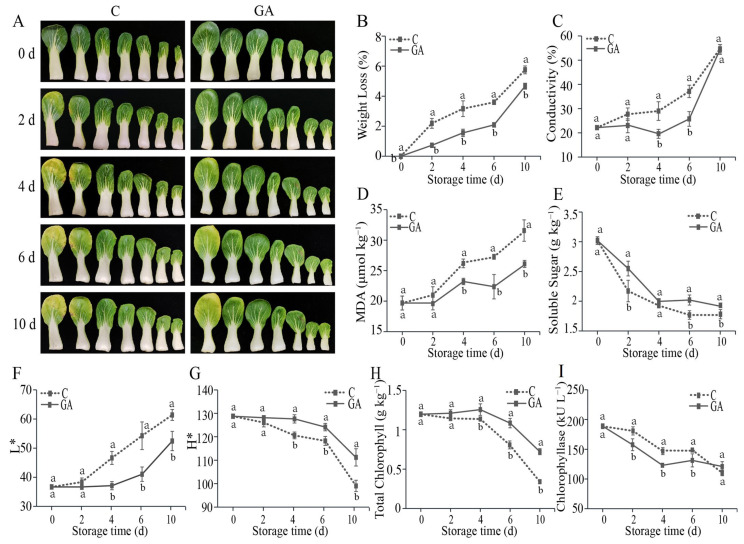
Effects of GA treatment on pak choi quality during storage. (**A**) Representative images; (**B**) Weight loss; (**C**) Conductivity; (**D**) MDA content; (**E**) Soluble sugar content; (**F**) L* value; (**G**) H* value; (**H**) Chlorophyll content; (**I**) Chlorophyllase activity. Values are the means ± SD (n = 3 biological replicates). Different lowercase letters indicate significant differences (*p* < 0.05).

To evaluate the impact of GA treatment on the quality of pak choi during storage, at the onset of this study, we commenced by applying varying concentrations of GA: 25 mg L^−1^, 50 mg L^−1^, and 100 mg L^−1^. After five days, the measurements of chlorophyll and MDA levels revealed that the leaves treated with a concentration of 100 mg L^−1^ exhibited a higher chlorophyll content and a lower MDA content in comparison to the control group (Figure S1). To further verify the effect of GA treatment on the quality of pak choi during storage, several key physiological indicators, including weight loss, leaf color (L* and H* parameters), cell membrane permeability (conductivity), lipid peroxidation (MDA content), nutritional quality (soluble sugars), total chlorophyll content, and chlorophyllase activity of 100 mg L^−1^ GA-treated pak choi were measured. Throughout the entire storage duration, the weight of pak choi exhibited a gradual decline. Notably, the treatment was 19% lower than the control on day 10 (Figure 1B). Compared with the control group, GA treatment decreased the conductivity on days 4 and 6 (Figure 1C). MDA levels exhibited an increase during leaf senescence, but levels of GA treatment were lower than control starting from day 4, and were 18% lower than control on day 10 (Figure 1D). For soluble sugar, the content gradually decreased after harvest, and GA treatment inhibited this reduction (Figure 1E).

Leaf yellowing represents the primary alteration observed in postharvest pak choi. As shown in Figure 1A, the leaves of pak choi subjected to GA treatment exhibited reduced yellowing in comparison to the control. The L^*^ value of the control samples increased steadily after 2 d of storage, while the treatment group increased after 4 d and was lower than the control group at each sampling point, and was 8.95 lower than the control group at 6 d (Figure 1F). The H^*^ value decreased after 4 d of storage, and GA inhibited the downregulation, which was 12.3 higher than control at 10 d (Figure 1G). The chlorophyll content exhibited a decline starting on day 4 of storage, while the application of GA sustained levels of chlorophyll. Finally, the chlorophyll content of the treatment was 2.12 times that of the control on day 10 (Figure 1H). Chlorophyllase activity decreased gradually in both groups during the storage period (Figure 1I).

### 3.2. Analysis of Differentially Expressed Genes (DEGs)

**Figure 2:**
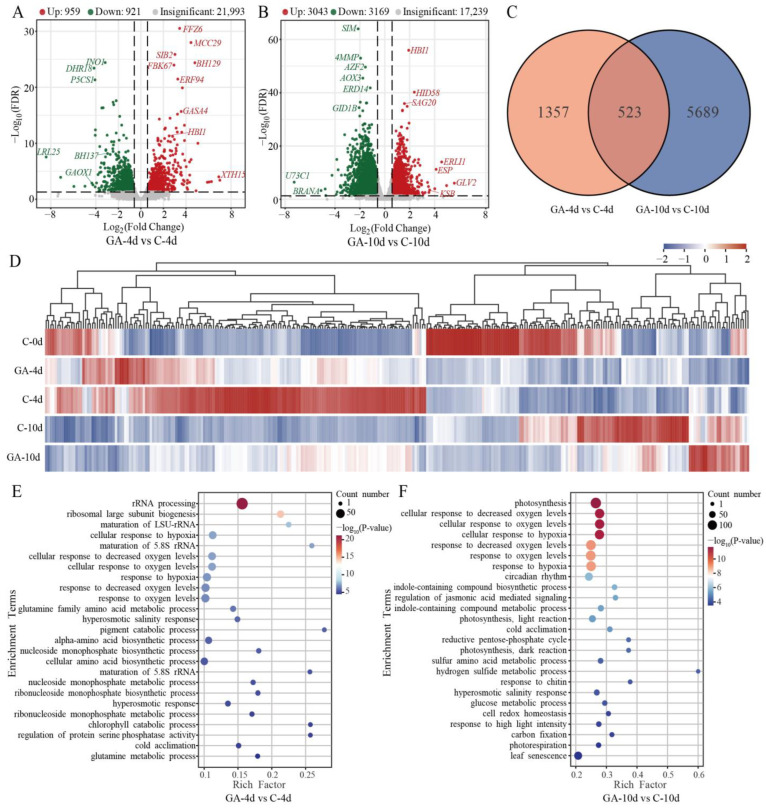
Differentially expressed genes (DEGs) in GA-treated (GA) and control (C) postharvest pak choi. (**A**) Volcano plots show the upregulated and downregulated DEGs in GA compared to the control at 4 d of storage. (**B**) Volcano plots show the upregulated and downregulated DEGs in GA compared to the control at 10 d of storage. Upregulated genes are represented by red dots and downregulated genes by green dots. (**C**) Venn diagram illustrating the overlap of DEGs at the 4 d and 10 d time points. (**D**) Heatmaps showing the expression levels of DEGs that were regulated at both 4 and 10 d of storage. (**E**) The top GO terms enriched for biological processes in the comparisons between GA-4d and C-4d. (**F**) The top GO terms enriched for biological processes in the comparisons between GA-10d and C-10d.

To elucidate the key regulatory genes underlying the observed variations in postharvest pak choi in response to GA treatment, RNA-seq was conducted on leaf samples collected at 0, 4, and 10 days of storage. A total of 20,988 genes exhibited changes in expression during the storage period of postharvest pak choi (Table S1). Among these, 5928 genes were upregulated and 6851 genes were downregulated in untreated pak choi after 4 days of storage. Similarly, 6450 genes were upregulated and 7970 genes were downregulated after 10 days of storage (Figure S2). A total of 7569 DEGs were identified in the comparison between GA-treated and non-treated groups. Of these, 959 and 3043 genes were upregulated at 4 and 10 d, respectively, while 921 and 3169 genes were downregulated at 4 and 10 d, respectively (Figure 2A,B). Venn diagram analysis identified 523 DEGs that were consistently regulated at both 4 and 10 days, providing critical insights into the temporal dynamics of GA-responsive gene expression (Figure 2C). This analysis enabled the identification of key candidate genes potentially associated with the regulation of postharvest senescence processes in pak choi.

To further explore the biological functions of the DEGs identified in GA-treated and control postharvest pak choi, GO and KEGG annotation analysis was conducted. The GO terms enriched for biological processes revealed that a substantial number of DEGs were associated with photosynthesis and response to oxygen levels. The enrichment of DEGs in terms related to chlorophyll catabolic metabolism and leaf senescence was particularly noteworthy, indicating that GA treatment impacts these crucial postharvest processes (Figure 2E,F). KEGG enrichment analysis revealed that genes involved in the plant hormone signal transduction pathway were notably enriched, with a total of 104 DEGs identified in the comparison between GA-4d and C-4d (Figure S3A). In the comparison between GA-10d and C-10d, DEGs were also found to be enriched in pathways associated with photosynthesis (Figure S3B). Overall, the functional enrichment analyses highlighted the important roles of photosynthesis, plant hormone signal transduction, leaf senescence, and chlorophyll metabolism in the regulation of postharvest leaf quality under GA treatment.

### 3.3. Regulation of Genes Related to Gibberellin, Chlorophyll, and Leaf Senescence

**Figure 3:**
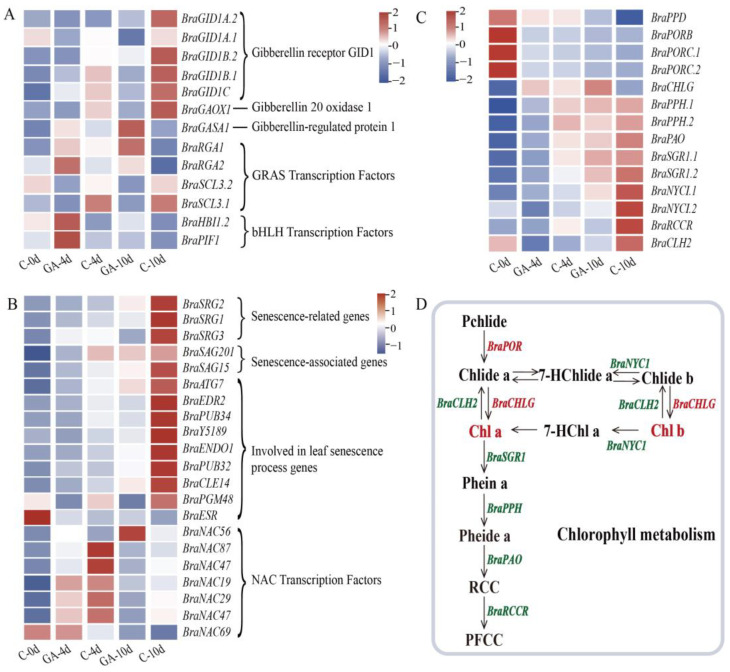
Impact of exogenous GA on gibberellin, senescence, and chlorophyll-related genes in postharvest pak choi. (**A**) Expression patterns of DEGs related to gibberellin in postharvest pak choi at 4 and 10 d of storage. (**B**) Expression patterns of DEGs involved in the leaf senescence process in postharvest pak choi at 4 d and 10 d of storage. (**C**) Expression patterns of DEGs of chlorophyll metabolism in postharvest pak choi at 4 and 10 d of storage. (**D**) Regulation of exogenous GA on the chlorophyll metabolism pathway. Red text indicates upregulation and green text indicates downregulation. Pcihlide: Protochlorophyllide, Phein a: Pheophytin a, Pheide a: Pheophorbide a, RCC: Red Chlorophyll Catabolite, PFCC: Primary Fluorescent Chlorophyll Catabolite, 7-HChlide a: 7-Hydroxy-Chlorophyllide a, 7-HChl a: 7-Hydroxy-Chlorophyll a, Chlide: Chlorophyllide, Chl: Chlorophyll.

As shown in Figure 3A, the results indicated that several genes related to gibberellin were regulated by exogenous GA, such as downregulation of the genes encoding GID1-like proteins (*BraGID1A*, *BraGID1B*, and *BraGID1C*). *BraGASA1* was upregulated by exogenous GA, which encodes a GAST1 protein homolog and negatively regulates the ripening process of fruit [20]. Exogenous GA inhibited gibberellin 20 oxidase 1 (*BraGAOX1*), a key gene for gibberellin biosynthesis. In addition, the *PIF4* transcription factor is regulated by exogenous GA [21], and the combined RGA and SCL transcription factors regulate GA signal transduction [22,23]. In our experiment, exogenous gibberellin affected the expression patterns of these genes.

The experimental findings identified 15 genes associated with leaf senescence from the DEGs. These comprise five senescence marker structural genes, senescence-related genes (*SRGs*), and senescence-associated genes (*SAGs*). Eight other structural genes and seven *NAC* transcription factors are also involved in the leaf senescence process. Specifically, the expression levels of *BraSRG1*, *BraSRG2*, *BraSRG3*, *BraSAG201*, and *BraSAG15* were downregulated by GA treatment compared with the control (Figure 3B). Other structural genes involved in the leaf senescence process also were downregulated by GA treatment (Figure 3B).

Chlorophyll degradation is a complex biochemical process that involves a series of structural genes and regulatory factors. As shown in Figure 3C, *BraNYCI* (Non-Yellow Coloring 1), *BraSGR1* (Stay-Green 1), *BraPPH* (Pheophytinase), *BraPAO* (Pheophorbide a oxygenase), and *BraRCCR* (Red chlorophyll catabolite reductase) are key genes responsible for the degradation of chlorophyll. Transcriptional analysis revealed that the expression levels of these genes gradually increased over the 4 d and 10 d storage periods in the control group, promoting chlorophyll degradation (Figure 3C). Upregulation of these genes was suppressed in the GA-treated samples (Figure 3C,D). In addition to the genes involved in chlorophyll degradation, several genes involved in chlorophyll biosynthesis were also examined. *BraPOR* (protochlorophyllide reductase) and *BraCHLG* (chlorophyll synthase) were upregulated in the GA-treated compared with the control. (Figure 3C).

### 3.4. Differential Accumulated Metabolites (DAMs) Analysis

**Figure 4:**
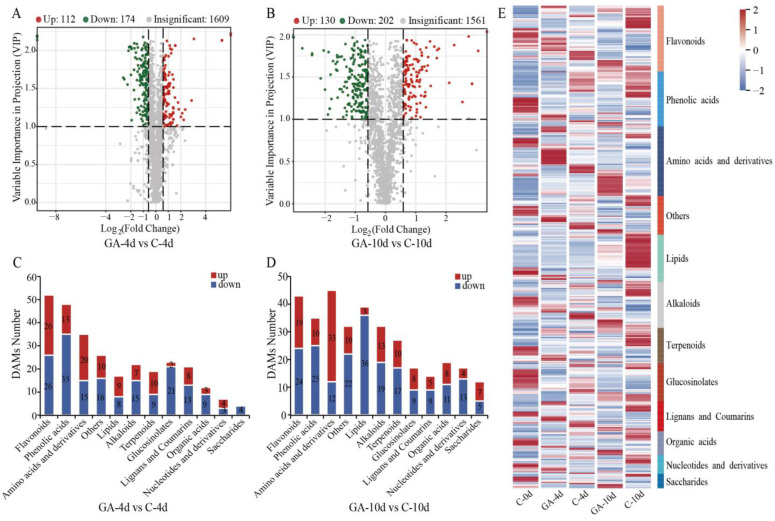
Differentially accumulated metabolites (DAMs) in GA-treated (GA) and control (**C**) postharvest pak choi. (**A**) Volcano plots show the upregulated and downregulated DAMs in GA compared to the control at 4 d of storage. (**B**) Volcano plots show the upregulated and downregulated DAMs in GA compared to the control at 10 d of storage. Upregulated metabolites are represented by red dots and downregulated genes by green dots. (**C**) The DAMs number of various categories in GA compared to the control at 4 d of storage. (**D**) The DAMs number of various categories in GA compared to the control at 10 d of storage. (**E**) Overview of the abundance pattern of DAMs in GA and control at 4 d and 10 d of storage.

To further elucidate the molecular mechanisms underlying the postharvest physiology of pak choi, a widely targeted metabolomic approach was employed to investigate the impact of exogenous GA on metabolite abundance. A total of 1446 metabolites accumulated differently, and GA treatment significantly altered the abundance of 546 DAMs during storage (Table S2). More metabolites accumulated than were reduced during storage (Figure S4). At 4 d, compared to the control, GA treatment resulted in the upregulation of 112 metabolites and the downregulation of 174 metabolites (Figure 4A). The predominant categories of these metabolites included flavonoids, amino acids and their derivatives, and phenolic acids (Figure 4C). On day 10, a total of 130 metabolites were upregulated, and 202 metabolites were downregulated in the GA-treated leaves (Figure 4B). Similar to the 4 d results, flavonoids, amino acids, and lipids were the most abundant metabolite categories identified (Figure 4D). Notably, certain metabolites in the leaf tissues exhibited distinct expression patterns following GA treatment (Figure 4E).

### 3.5. Regulation of Lipid Metabolism by Exogenous GA During Leaf Senescence

**Figure 5:**
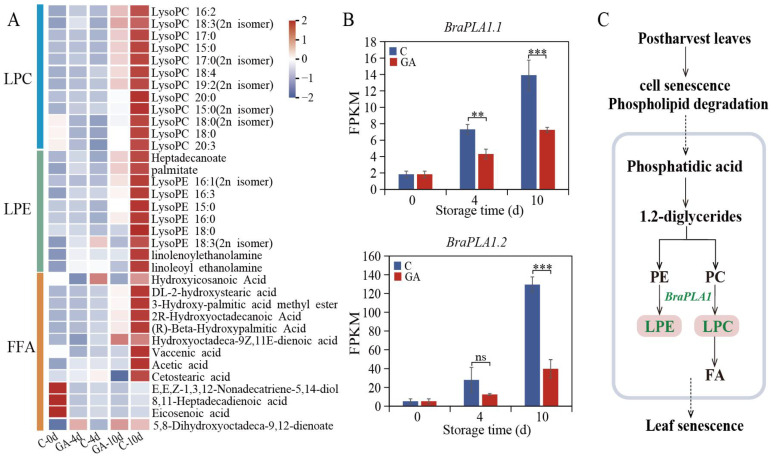
Modulation of exogenous GA on lipid metabolism in postharvest pak choi. (**A**) Heatmaps showing the levels of lipid DAMs in postharvest pak choi at 4 and 10 d of storage. (**B**) Expression levels of *BraPLA1* during leaf senescence in control and GA-treated pak choi. Symbols **, and *** denote a significant difference at *p* < 0.01, and *p* < 0.001, respectively. (**C**) Regulation of exogenous GA on the phospholipid metabolism pathway. Green text indicates downregulation. LPE: lysophosphatidylethanolamine, LPC: lysophosphatidylcholine, FFA: free fatty acids, FA: fatty acids, PE: phosphatidylethanolamine, PC: phosphatidylcholines.

The alterations in composition and concentration of lipids play a crucial role in the process of leaf senescence, including membrane degradation, energy mobilization, and cellular breakdown. The application of GA has been shown to modulate the trajectory of lipid changes during senescence. In this study, a total of 39 lipid metabolites were found to be influenced by GA treatment. Notably, 36 out of these 39 lipid metabolites exhibited a lower abundance than the control after GA treatment. GA suppresses the upregulation of lipid metabolites typically observed during senescence. These lipid metabolites mostly consist of lysophosphatidylcholine (LPC), lysophosphatidylethanolamine (LPE), and free fatty acids (FFA) (Figure 5A). In addition, Phospholipase A (*PLA*) catalyzes the hydrolysis of phospholipids, leading to the formation of LPC, LPE, FFA, and other lipid derivatives [24]. Transcriptomic analysis revealed an upregulation of *BraPLA1* during leaf senescence, which was subsequently inhibited by GA treatment (Figure 5B). This suppression of *BraPLA1* expression was observed along with a reduction in the levels of LPC and LPE in GA-treated leaves (Figure 5C).

### 3.6. Regulation of Jasmonic Acid Signal Pathway by Exogenous GA During Leaf Senescence

**Figure 6:**
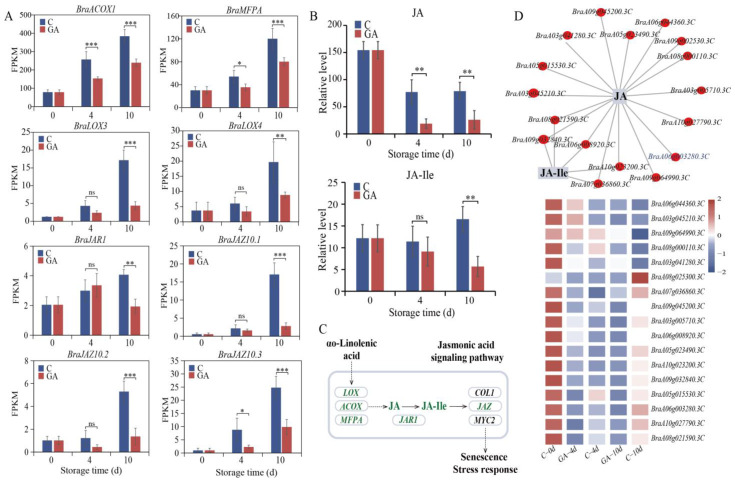
Regulation of exogenous GA on the jasmonic acid signal pathway in postharvest pak choi. (**A**) Expression levels of *BraACOX1*, *BraMFPA*, *BraLOX*, *BraJAR1*, and *BraJAZ10* during leaf senescence in the control and GA-treated pak choi. (**B**) The abundance of JA and JA-Ile in control and GA-treated pak choi during storage. Symbols *, **, and *** denote a significant difference at *p* < 0.05, *p* < 0.01, and *p* < 0.001, respectively. (**C**) Regulation of exogenous GA on the JA signal pathway. Green text indicates downregulation. JA: Jasmonic acid, JA-Ile: Jasmonate-Isoleucine. (**D**) The network and expression patterns of DEGs exhibit significant correlations with the abundance of JA and JA Ile.

Jasmonic acid (JA) is a key plant hormone that plays a pivotal role in regulating leaf senescence [25]. GA treatment effectively suppresses the upregulation of both JA and jasmonate-isoleucine (JA-Ile) during the postharvest senescence of pak choi leaves (Figure 6B). In particular, six genes encoding enzymes involved in the metabolism of JA were identified, including jasmonate ZIM-domain proteins (*BraJAZ*), jasmonate-L-amino acid synthase (*BraJAR1*), and JA synthesis-related genes (*BraLOX*, *BraACOX1*, and *BraMFPA*). After GA treatment, the expression of these genes was downregulated compared to the control during storage (Figure 6A). These regulated metabolites and genes were involved in the JA signal transduction pathway (Figure 6C). The integrated analysis of transcriptomic and metabolomic data revealed that an additional 17 DEGs exhibited strong correlations with JA and JA-Ile levels (Figure 6D). Notably, the suppression of JA and gene expression in GA-treated leaves suggests that GA treatment interferes with the JA-mediated senescence pathways.

## 4. Discussion

Pak choi is an important leafy vegetable in China, with extensive cultivation and substantial annual yields. Nevertheless, leafy vegetables lack nutritional supply after harvest, with appearance changes such as yellowing and wilting of leaves, physiological changes such as nutrient loss, and exacerbation of lipid peroxidation [26]. GA treatment has been demonstrated to be an effective postharvest technology for inhibiting ripening, preserving quality, and delaying the senescence of fruits and vegetables [9,10,14,27]. However, the impact of GA on the postharvest storage of *Brassica rapa* remains unclear. In this study, 100 mg L^−1^ GA was administered to postharvest pak choi, and the underlying mechanisms of GA treatment on postharvest pak choi were explored through transcriptome and metabolome analyses.

### 4.1. GA Treatment Delays Leaf Senescence in Postharvest Pak Choi

Our results demonstrate that GA treatment effectively delays postharvest leaf senescence in pak choi by modulating the expression of senescence-associated genes and altering key metabolic pathways. During postharvest storage, physiological changes in fruits and vegetables include weight reduction, increased conductivity [28], elevated levels of malondialdehyde [29], decreased soluble sugar content [30], and reduction in the chlorophyll content of green vegetables [31]. Following GA treatment, the onset of the aforementioned senescence-related changes was delayed (Figure 1). Molecularly, the senescence process is governed by numerous senescence-associated structural genes and transcription factors, including *SAGs*, *SRGs*, the *NAC* transcription factor [32], and the *WRKY* transcription factor [33]. Research has demonstrated that the expression of *AtSAG20* in *Arabidopsis* is associated with the gradual progression of leaf senescence [4,34]. The expression of *BraSAG201* and *BraSAG15* was modulated following GA treatment, with their upregulation during senescence being significantly suppressed (Figure 3B). Additionally, it has been discovered that the *NAC* transcription factor *BrNAC029* in Chinese cabbage plays a role in delaying leaf senescence in response to exogenous cytokinin [32]. Our study revealed that seven *NAC* transcription factors were regulated in response to gibberellin treatment. Notably, comparative expression profiling demonstrated that GA application resulted in the downregulation of *BraSRG1*, *BraSRG2*, and *BraSRG3* on day 10 compared to untreated controls. These molecular changes were associated with delayed postharvest senescence in pak choi, as evidenced by phenotypic observations (Figure 3B).

### 4.2. GA Treatment Inhibits Chlorophyll Degradation Pathways

Chlorophyll degradation constitutes the primary mechanism underlying postharvest senescence in leafy vegetables and is modulated by several important genes, including *NYC1*, *SGR1*, *PPH*, *PAO*, and *RCCR* [35]. This is a complex reaction system, wherein chlorophyll a is decomposed to yield pheophytin a, which is also the first step of chlorophyll degradation [36,37]. The application of GA resulted in the suppression of chlorophyll catabolic gene expression, including *BrPPH*, *BrPAO*, *BrSGR1*, and *BrNYC1*, and preserved elevated total chlorophyll levels throughout postharvest storage, thereby delaying the onset of leaf senescence [14,38]. In our study, consistent with prior findings, postharvest pak choi subjected to exogenous GA exhibited minimal color alteration during storage (Figure 1F,G). The chlorophyll content was maintained, and chlorophyllase activity was notably low (Figure 1H,I). Transcriptomic analysis revealed that GA downregulated key genes at every stage of chlorophyll degradation, including *BraNYCI*, *BraCLH*, *BraSGR1*, *BraPPH*, *BraPAO*, and *BraRCCR*. Notably, GA treatment led to a marked upregulation of two structural genes, *BraPOR* and *BraCHLG*, involved in chlorophyll synthesis (Figure 3D). This upregulation was consistent with the observed pattern of chlorophyll accumulation in GA-treated leaves compared to the control group. These experimental findings provide substantial evidence that the application of GA inhibited the degradation of chlorophyll in postharvest pak choi, which is an important mechanism by which GA mitigates postharvest senescence in pak choi.

### 4.3. GA Treatment Modulates Lipid Metabolism and Stabilizes Membrane Integrity

Phospholipids are fundamental components that ensure the structural dynamics and integrity of cellular membranes [39]. Phosphatidylcholine (PC), a phospholipid that contains choline, plays a pivotal role in cellular integrity. Upon cellular membrane disruption, phospholipase A catalyzes the hydrolysis of PC, yielding LPC [24]. LPC exerts significant biological functions as a secondary messenger that modulates cellular processes such as growth, differentiation, and apoptosis. LPC could induce beta-galactosidase (*GLB1*), a biomarker indicative of cellular senescence. This induction can result in an elevated production of reactive oxygen species (ROS), which leads to DNA damage and contributes to cellular transformation and carcinogenesis [40]. In our experiment, GA treatment influenced the trend of lipid composition and content changes, and downregulated various decomposition products of phospholipids, including LPC, in senescent pak choi (Figure 5A). Furthermore, the expression patterns of the genes encoding phospholipase A, *BraPLA1.1* and *BraPLA1.2*, were modulated by GA treatment. Notably, their transcript levels were markedly downregulated compared to the control (Figure 5B). Those results suggest that GA treatment effectively modulates lipid metabolism, particularly the degradation of membrane phospholipids, and delays senescence in postharvest pak choi by stabilizing cellular membrane integrity. However, the underlying regulatory mechanisms of the lipid pathway involved in this process require further investigation.

### 4.4. GA Interferes with JA-Mediated Senescence Pathways

Plant hormones play a crucial role in regulating leaf senescence, which includes positive regulators such as ethylene, abscisic acid, and jasmonic acid, and negative regulators, including auxin, gibberellin, and cytokinins [41,42,43]. JA is a lipid-derived plant hormone that has been implicated in the signal pathways controlling leaf senescence [44]. An increasing body of evidence indicates that the transcriptional abundance of genes involved in JA signal transduction, such as *COL1*, *MYC2*, *JAZ*, and *LOX*, increases with leaf senescence [45,46,47]. In our study, the gene expression related to the JA signal transduction pathway, including *BraJAZ10*, *BraJAR1*, *BraLOX*, *BraACOX1*, and *BraMFPA*, was lower in GA-treated samples (Figure 6A). Furthermore, the increase in JA-Ile (the bioactive form of JA) was also curtailed by GA treatment. While endogenous JA levels exhibited a declining trend during storage, this reduction was further accentuated by GA application (Figure 6B). Research has demonstrated that the application of JA upregulates the transcriptional levels of senescence-associated genetic marker genes, such as *SAGs* [44]. Furthermore, in *Arabidopsis* mutants defective in all JA responses, 12% of the genes associated with development and senescence ceased to be upregulated [48]. In our study, JA signal transduction was inhibited while the concurrent upregulation of *SAGs* was inhibited. This illustrates that exogenous GA might suppress the JA signal pathway, potentially delaying the senescence of postharvest pak choi. The application of JA facilitates the degradation of chlorophyll, expedites lipid peroxidation and membrane degradation, and finally accelerates the senescence process in the flag leaves of rice [49]. It is reasonable to believe that GA and JA have antagonistic effects on the leaf senescence process in pak choi. However, further research into the underlying regulatory processes is required to fully understand these relationships.

Ethylene (ET) is also a plant hormone. JA and ET exhibit synergistic effects in senescence and stress responses, while GA and ET have antagonistic interactions during plant senescence [50,51]. Studies have shown that GA regulates fruit development by modulating the expression of ethylene-responsive transcription factor (*ERF*) genes in tomatoes, thereby influencing the ethylene signaling pathway [52]. In our experiments, transcriptomic analysis revealed that GA treatment affected the expression of 35 *ERFs*, with 14 of these genes being downregulated in the later stages of storage (Figure S5). This suggests that GA may modulate the ethylene signaling pathway, potentially contributing to the delayed senescence observed in GA-treated pak choi. The downregulation of *ERFs* aligns with previous studies showing that GA and ET often exhibit antagonistic interactions during plant senescence. In conclusion, our findings suggest that GA not only inhibits JA-mediated senescence but also influences ethylene-responsive genes. This highlights the complex regulatory network involving GA, JA, and ET in postharvest senescence.

GA treatment affects chlorophyll degradation, phospholipid metabolism, membrane integrity, and the regulation of senescence by plant hormones, such as JA in postharvest pak choi. These findings suggest that GA treatment could serve as a viable strategy to delay senescence and enhance postharvest quality, thereby extending the shelf life of leafy vegetables under commercial storage and transportation conditions. To translate these findings into practical applications, further investigations are required to optimize GA treatment protocols for commercial storage and transport conditions. Further exploration of the underlying mechanisms and ensuring its efficacy is necessary, as well as a focus on fine-tuning GA concentrations and investigating their interactions with other postharvest treatments.

## 5. Conclusions

In conclusion, this study demonstrates that GA treatment effectively delays postharvest senescence in pak choi by modulating key physiological and molecular pathways. The results reveal that GA treatment suppressed the expression of senescence-related genes, including *BraSRG1*, *BraSGR2*, *BraSGR3*, *BraSRG201*, and *BraSRG15*. It also maintained chlorophyll content by modulating genes involved in chlorophyll metabolism, such as *BraPPH*, *BraSGR1*, *BraNYCI*, and *BraPAO*. Additionally, GA treatment influenced lipid levels, effectively modulating the degradation of membrane phospholipids. The jasmonic acid signaling pathway was repressed by GA. These findings enhance our understanding of the role of GA in postharvest senescence and provide valuable insights for developing strategies to improve the postharvest quality of leafy vegetables.

## Data Availability

The original contributions presented in the study are included in the article, further inquiries can be directed to the corresponding author.

## Supplementary Materials

The following supporting information can be downloaded at: https://www.mdpi.com/article/10.3390/foods14060981/s1, Figure S1: Total chlorophyll (A) and malondialdehyde (MDA) content (B) in pak choi treated with 0 mg L^−1^, 25 mg L^−1^, 50 mg L^−1^, and 100 mg L^−1^ gibberellic acid after 5 days of storage; Figure S2: Numbers of upregulated and downregulated differentially expressed genes (DEGs) in pak choi under control and GA treatments at 4 days and 10 days of storage; Figure S3: KEGG pathway enrichment analysis of differentially expressed genes (DEGs) in pak choi at 4 days (A) and 10 days (B) of storage; Figure S4: Numbers of upregulated and downregulated differentially accumulated metabolites (DAMs) in pak choi under control and GA treatments at 4 days and 10 days of storage; Figure S5: Expression patterns of ethylene-responsive transcription factors in pak choi under control and GA treatments at 4 days and 10 days of storage; Table S1: All DEGs of GA vs C at 4 d and 10 d of storage; Table S2: All DAMs of GA vs C at 4 d and 10 d of storage.
