# Supplementary figures and images for: Exogenous Gibberellin Delays Postharvest Leaf Senescence in Pak Choi by Modulating Transcriptomic and Metabolomic Profiles
Source: Foods. 2025 Mar 13;14(6):981. doi: 10.3390/foods14060981 (PMC11941532; doi:10.3390/foods14060981)

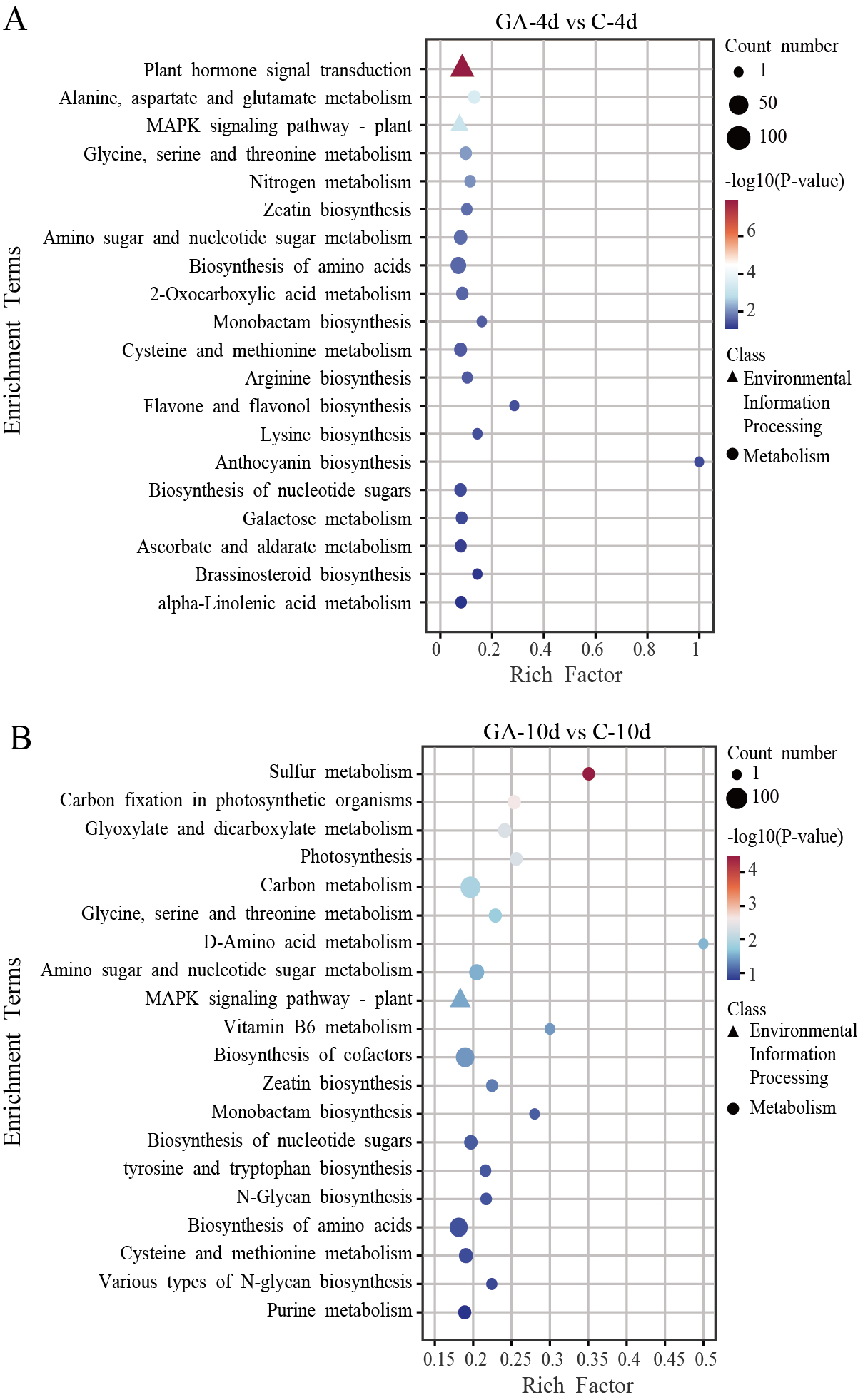

Supplement: Supplementary file 1 [file foods-14-00981-s001.zip › FigureS3.jpg]

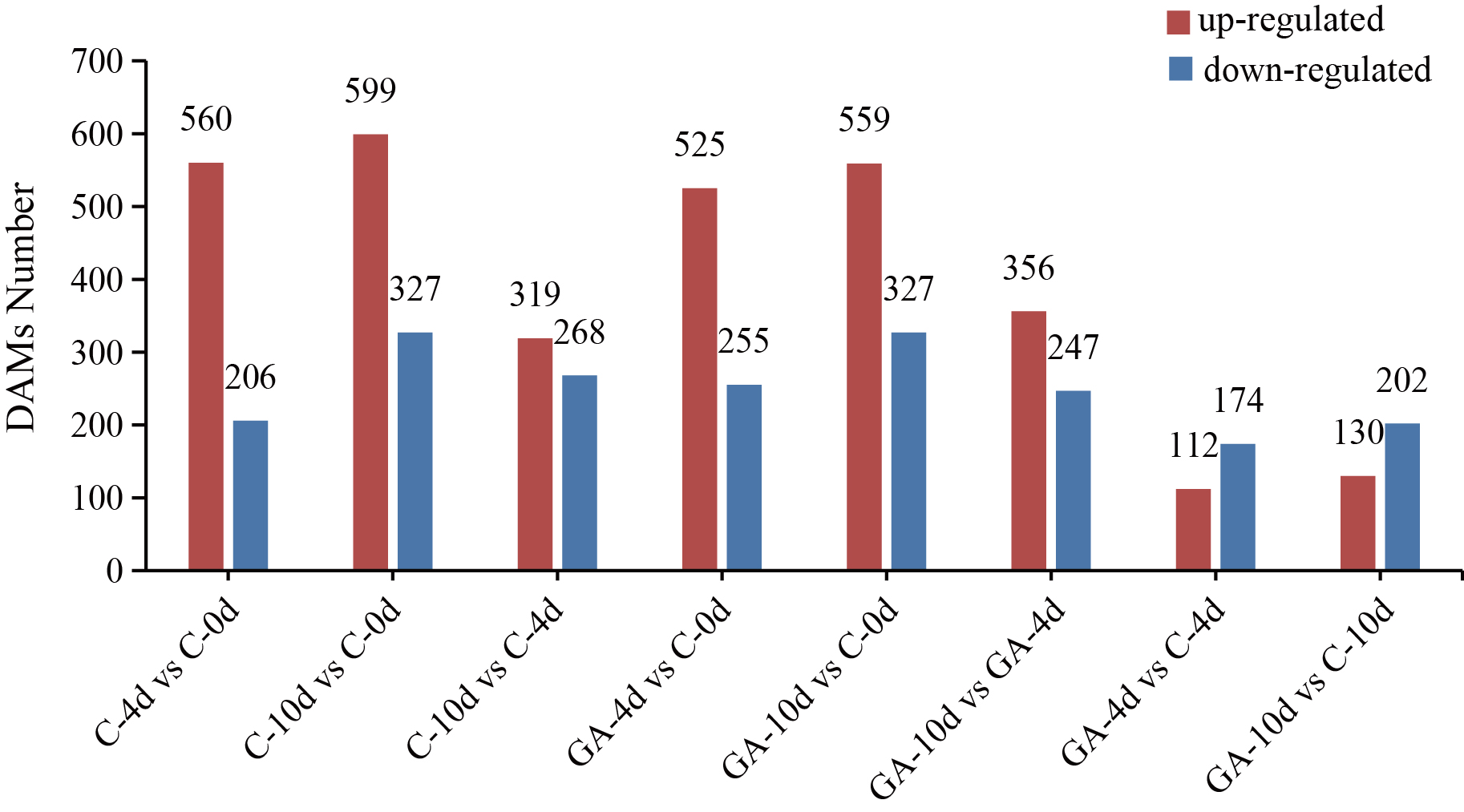

Supplement: Supplementary file 1 [file foods-14-00981-s001.zip › FigureS4.jpg]

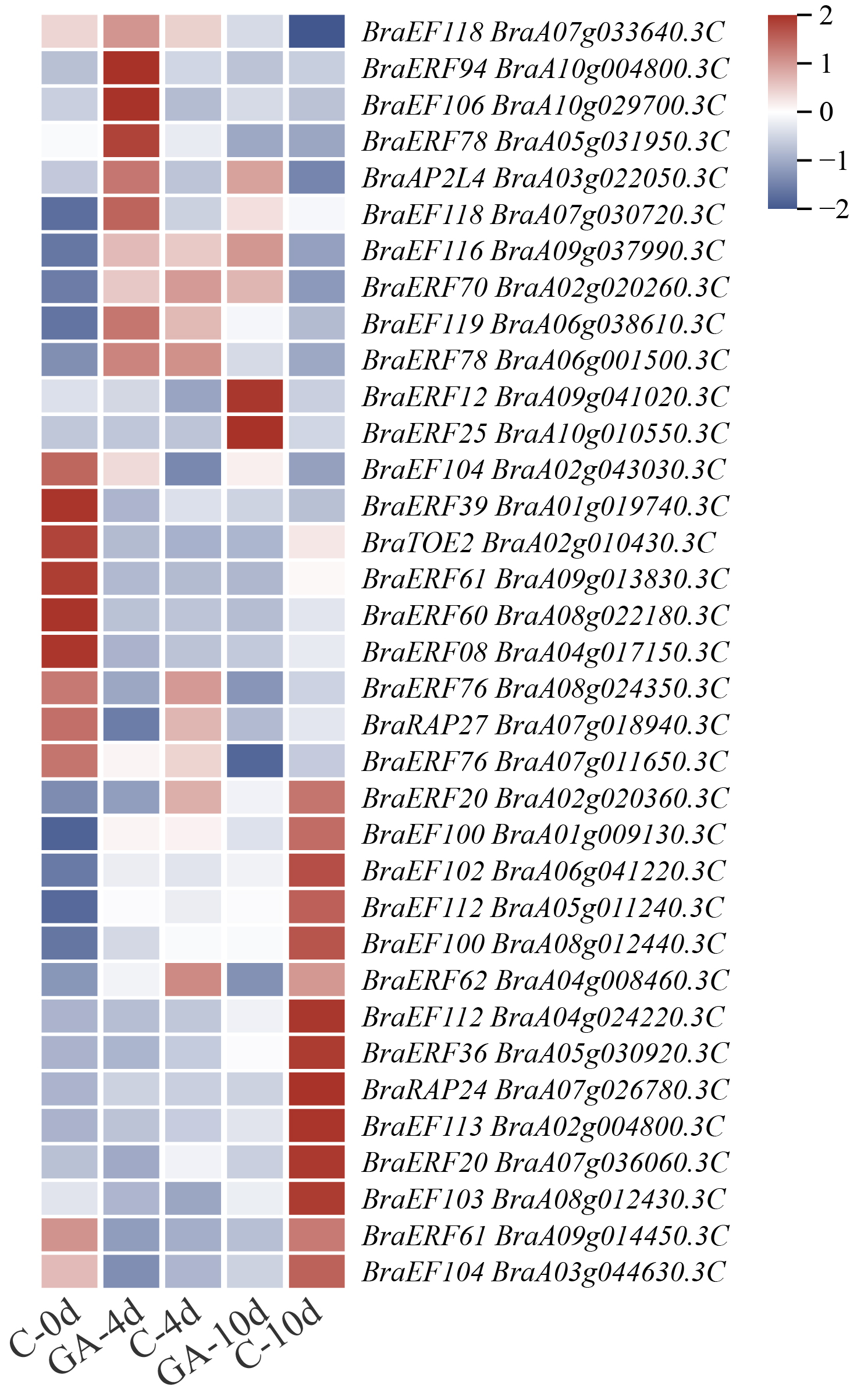

Supplement: Supplementary file 1 [file foods-14-00981-s001.zip › FigureS5.jpg]

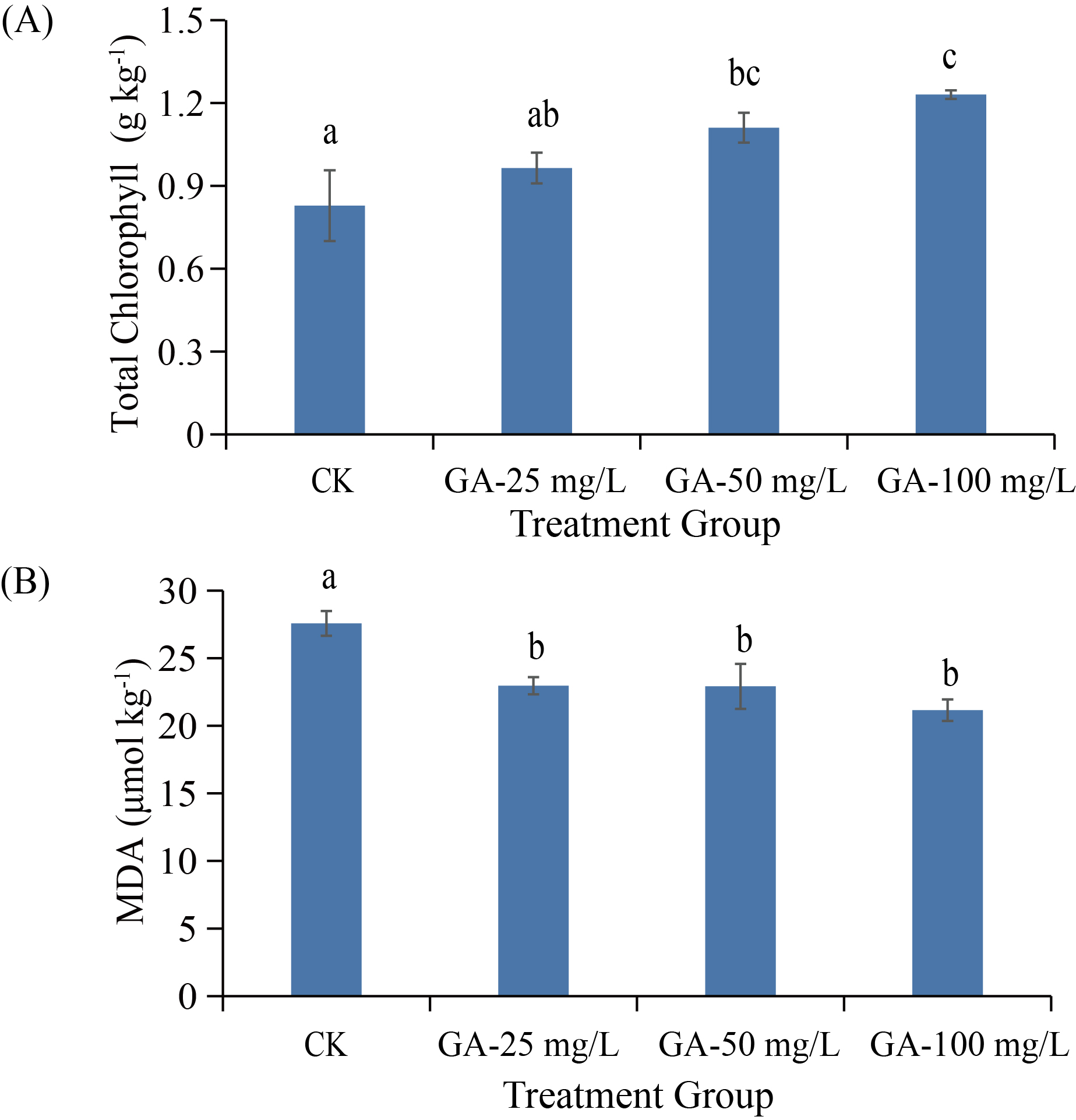

Supplement: Supplementary file 1 [file foods-14-00981-s001.zip › FigureS1.jpg]

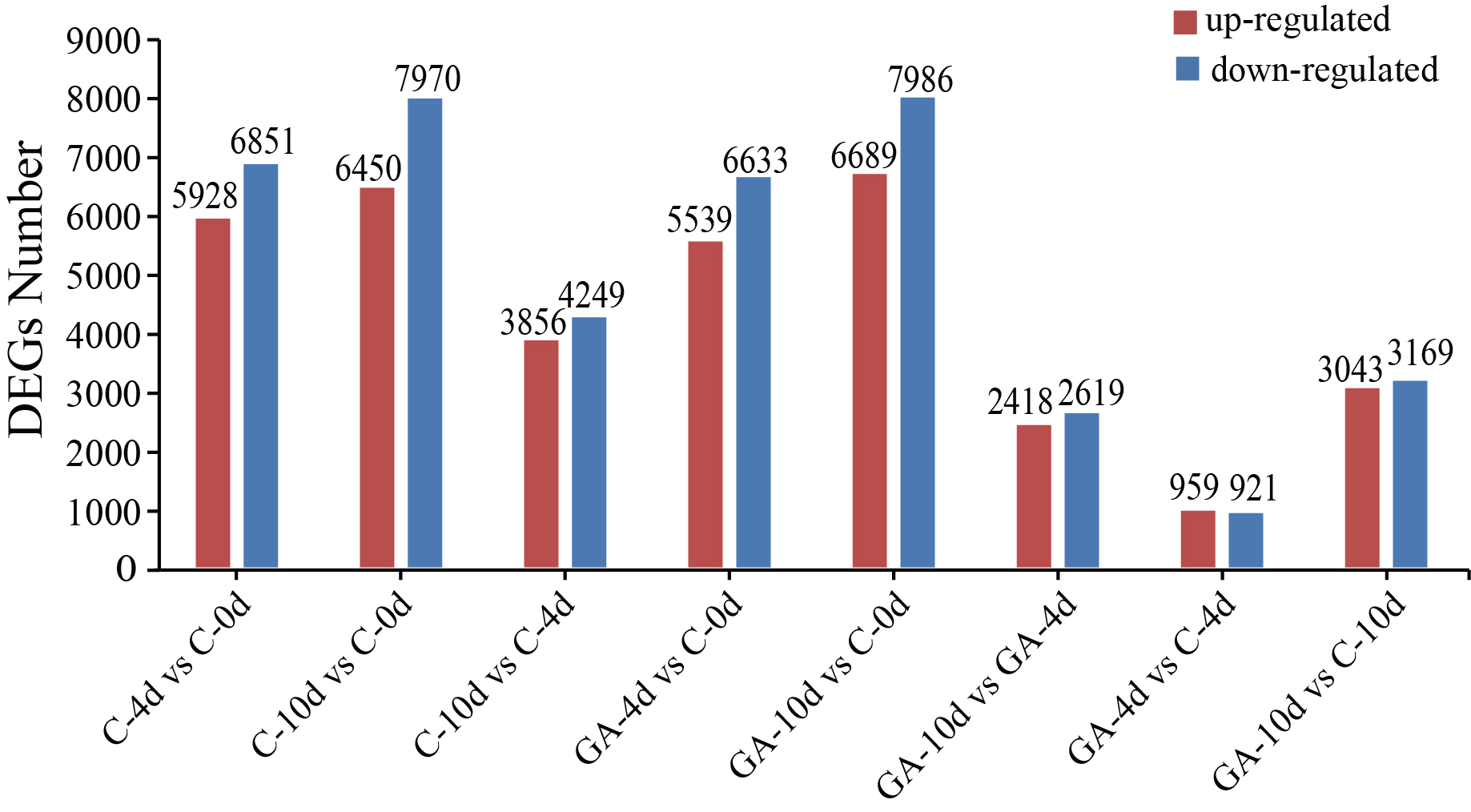

Supplement: Supplementary file 1 [file foods-14-00981-s001.zip › FigureS2.jpg]
